# Is there a nitrogen fertilizer threshold emitting less N_2_O with the prerequisite of high wheat production?

**DOI:** 10.1371/journal.pone.0202343

**Published:** 2018-08-28

**Authors:** Yuan Yi, Fujian Li, Mingwei Zhang, Yi Yuan, Min Zhu, Wenshan Guo, Xinkai Zhu, Chunyan Li

**Affiliations:** 1 Laboratory of Crop Genetics and Physiology of Jiangsu Province / Co-Innovation Center for Modern Production Technology of Grain Crops / Wheat Research Institute, Yangzhou University, Yangzhou, China; 2 Zhenjiang Agro-Technical Extension Station No. 97, Zhenjiang City, Jiangsu Province, China; Tennessee State University, UNITED STATES

## Abstract

Excessive use of synthetic nitrogen (N) fertilizer and lower nitrogen use efficiency (NUE) are threatening the wheat production in the middle and lower reaches of Yangtze River. Excess input of N fertilizers also results in severe environmental pollution, climate change and biodiversity loss. However, the study on reasonable nitrogen application and NUE improvement with the prerequisite of stable and high yield remains unexplored. In our study, the four different levels of nitrogen were applied to find out the nitrogen threshold which could be both friendly to environment and promise the stable and high yield. The experiment was carried out in Yangzhou University (Yangzhou, China). The wheat cultivar Yangmai 23 was selected as the research material. The four nitrogen levels were as follows: 0, 189, 229.5, and 270 kg ha^-1^. The results showed that the grain yield under the application of 229.5 kg ha^-1^ N was as high as that under 270 kg ha^-1^ N level, with the observation of 20.3% increase in agronomic efficiency. The N_2_O emission of 229.5 kg ha^-1^ N application was as low as that of 189 kg ha^-1^ N, but the grain yield and agronomic efficiency were significantly higher (11.9%) under 229.5 kg ha^-1^ treatment than the lower one. Taken together, this indicated the nitrogen level at 229.5 kg ha^-1^ could be identified as the fertilizer threshold, which will be beneficial for the future fieldwork.

## 1. Introduction

Wheat is a dominant crop used for human food and livestock feed in temperate countries [1]. China is the largest wheat producer in the world, with an annual sowing area of approximately 23.4 million ha and production of 105 million tons [2]. Due to the ubiquitous utilization of synthetic nitrogen (N) fertilizer, it is easier for China to feed 22% of the world’s population using only 9% of the available arable land [3]. The high input of synthetic N fertilizer has contributed to a substantial increase in wheat production in China [4]. Consequently, China has become the largest consumer of N fertilizer in the world, and over 30% of the world’s total consumption is used by China [5]. Based on field experiments and investigations, NARs have reached 270 kg ha^-1^ or more, which is much higher than suggested [6, 7]. However, the nitrogen use efficiency (NUE) of wheat cultivated in China is only between 24.8% and 35.7%, which is much lower than the typical level of 50% reported in most developed countries [8,9]. It is estimated that 1% increase in NUE could save about $1.1 billion annually [10]; hence, improving NUE is essential for the development of sustainable agriculture [11, 12].

Since the mechanisms underlying NUE are complicated [11], several nitrogen utilization parameters have been applied in previous papers to help grasp this complexity. Partial factor productivity (PFP_N_), the ratio of total grain output to applied N inputs, reflects the situation of incremental increase in yield that results from N application and the use efficiency of endogenous N resources absorbed by the plant [13]. Agronomic efficiency (AE_N_) is a method to estimate the efficiency of converting applied N to grain yield [12] and it is made up mainly of two physiological components, N apparent recovery efficiency (AR_N_) and N physiological efficiency (PE_N_) [12].

Due to the high N application rates (NAR) and the low NUE in China, a large portion of the N fertilizer is wasted and affects the environment around agricultural lands [14]. Apart from the contamination of ground and surface water, the N-related massive emission of greenhouse gas (GHG) and the consecutive contributions to global warming constitute a serious threat to crop production sustainability. It has been estimated that agriculture contributes approximately 84% and 52% of the global anthropogenic N_2_O and CH_4_ emissions, respectively [15], while it is only responsible for approximately 1% of CO_2_ emissions [16]. Due to the wide application of synthetic N since the pre-industrial era, the concentrations of CH_4_ and N_2_O in the atmosphere have increased by 148% and 18%, respectively [17]. Based on previous studies, the agricultural CH_4_ and N_2_O emissions are likely to increase by 60% over the next two decades because of these increasing N applications [18]. In China, the CH_4_ and N_2_O emissions from wheat fields were estimated to range from 7.4 to 8.0 kg CH_4_ year^-1^ and from 88.0 to 98.1 g N_2_O N year^-1^, respectively [19]. Zhang et al. estimated that approximately 7% of the GHG emissions from the entire Chinese economy are N-fertilizer-related emissions [20], while the contribution of synthetic fertilizer use to the total GHG emission from EU-15 countries is only approximately 2% [21]. Moreover, recent studies have shown that the potential greenhouse gas emissions associated with the agricultural N additions in the lower reaches of the Yangtze River are highest in China [22]; furthermore, anthropogenic soil acidification driven by N fertilization has significantly increased in rice-wheat double-cropping systems since the 1980s [23]. Hence, it is urgent to accommodate the needs of the expanding world population by developing highly productive agriculture; however, it is also necessary to simultaneously preserve the quality of the environment [24].

The middle and lower reaches of the Yangtze River, which have typical high-yield rice-wheat double-cropping systems, have important contributions to wheat production in China. However, the excessive use of synthetic nitrogen (N) fertilizer along with the lower nitrogen use efficiency (NUE) have become restraints for wheat production in this region. Using the appropriate NARs could help increase biomass production and decrease GHG emissions [25]. In field experiments, it has successfully been shown that significant reductions in the NAR and related environmental impacts are possible without significantly reducing the yield [26, 27]. A 20–25% reduction in the NAR in winter wheat, relative to present levels, is recommended in the southern part of China [28]. Accordingly, the important objectives of this study are as follows: (a) to determine whether it is possible to decrease the NAR from the conventional level used by local farmers (i.e., 270 kg N ha^-1^) by 15% or 30% without causing significant declines in yield while simultaneously reducing GHG emissions; (b) to measure the seasonal GHG emission as N_2_O and CH_4_; and (c) to investigate the correlation between the GHG emissions and nitrogen utilization parameters.

## 2. Materials and methods

A field experiment was conducted at the experimental station of Yangzhou University, China (32.39°N, 119.42°E). The site is located in the middle and lower reaches of the Yangtze River, which has a subtropical monsoon climate. The soil was a sandy-loam, and the soil properties (0–20 cm soil layer) were characterized using the methods previously described by Lu [29]. Before land preparation, composite soil samples (0–20 cm depth) were collected and analyzed using the methods described by Lu [29]. The soil contained 1.7% organic matter, 0.7 g kg^-1^ total N, 75.2 mg kg^-1^ available N, 54.8 mg kg^-1^ available P, and 181.2 mg kg^-1^ available K in the 2013/2014 growing season; additionally, the soil contained 1.3% organic matter, 0.6 g kg^-1^ total N, 67.2 mg kg^-1^ available N, 45.5 mg kg^-1^ available P, and 99.3 mg kg^-1^ available K in the 2014/2015 growing season. The main meteorological data from two wheat growing seasons were measured and are summarized in Table 1. The stages of wheat growing were classified and referred to previous study as follow [30]: sowing, over-wintering, jointing, stem elongation, booting, and maturing stages, which corresponded to 0, 41, 115, 129, 156 and 211 d after sowing, respectively, during the 2013/2014 growing season; and 0, 43, 120, 136, 162 and 213 d after sowing, respectively, during the 2014/2015 growing season.

**Table 1:** The main meteorological data from two wheat growing seasons.

Growth period	2013/2014				2014/2015			
	Days after sowing (d)	Effective accumulated temperature (>0°C)	Precipitation (mm)	Sunshine duration (h)	Days after sowing (d)	Effective accumulated temperature (>0°C)	Precipitation (mm)	Sunshine duration (h)
Sowing—Over-wintering	0–41	629	33	348	0–43	612	104	310
Over-wintering—Jointing	42–115	231	120	283	44–120	276	39	313
Jointing—Elongation	116–129	130	28	76	121–139	109	20	41
Elongation—Booting	130–156	593	182	232	140–162	518	140	209
Booting—Maturity	157–211	870	88	281	157–213	844	136	266
Total	-	2452	451	1220	-	2359	462	1139

### 2.1 Experimental design and field management

The field trial used a randomized complete block design with three replicates per treatment. Yangmai23, a locally adapted new cultivar with strong gluten, was planted and rotated with paddy rice in this experiment, and the cultivar was supplied by the Lixiahe Agricultural Research Institute of China. In the 2013/2014 growing season, the wheat was sown on October 28 and harvested on June 1. In the 2014/2015 growing season, the wheat was sown on November 2 and harvested on June 3. Each plot measured 7.5 m in length × 3 m in width and had a theoretical density of 225 seeds per m^2^. The four nitrogen levels were 0 (0N), 189 (LN), 229.5 (MN), and 270 (HN) kg N ha^-1^; moreover, the 30% and 15% reductions in the NAR (relative to the conventional NAR used by local farmers of 270 kg N ha^-1^) corresponded to 189 and 229.5 kg N ha^-1^, respectively. Fertilizers were applied as urea (nitrogen content of 46.3%). The fertilization was divided into four stages, including the before sowing, tillering, beginning of stem elongation and booting stages, which had 50%, 10%, 20% and 20% of the four designated fertilizer amounts, respectively. The four fertilization stages corresponded to 0, 38, 119, and 147 d after sowing, respectively, during the 2013/2014 growing season, and to 0, 39, 122, and 149 d after sowing, respectively, during the 2014/2015 growing season. For all treatments, 120 kg ha^−1^ P_2_O_5_ (calculated from super-phosphate) and 120 kg ha^−1^ K_2_O (calculated from potassium chloride) were applied before sowing to guarantee there was no stress related to the amount of phosphate and potassium [30].

### 2.2 Sampling and data collection

#### 2.2.1 Grain yield and N content

During the entire experiment, the dates of the key growth stages of crops were recorded. Wheat plants were harvested from 1m^2^ subplots to determine the number of effective spikes. The grain numbers per spike were counted from 50 selected spikes. Three samples were weighed to obtain the mean thousand-grain weight for each plot. All harvested samples were threshed, and the grain yield was standardized at 13% moisture content. The concentrations of N in grain and straw were determined by micro-Kjeldahl [31], followed by digestion in a H_2_SO_4_–H_2_O_2_ solution. The yield response was calculated as follows [32]:

$$\mathrm{Yield}\;\mathrm{response}\left({\mathrm{kg}\;\mathrm{ha}}^{‑1}\right){=Y}_{N}{‑Y}_{0}.$$

where Y_N_ is the grain yield (kg ha^-1^) at a certain level of applied N fertilizer, and Y_0_ is the grain yield (kg ha^-1^) without N application.

#### 2.2.2 Nitrogen utilization parameters

The calculations for the nitrogen utilization parameters were as follows [33, 34]:

$${\mathrm{AR}}_{N}\left(\mathrm{apparent}\;\mathrm{recovery}\;\mathrm{efficiency}\;\mathrm{of}\;\mathrm{applied}\;N\right)\;\left(\%\right)=\left(U_{N}{‑U}_{0}\right){/F}_{N}\times 100.$$

where U_N_ is the total N uptake (kg ha^-1^) in the shoot, U_0_ is the total N uptake measured without N application, and F_N_ is the rate of applied N fertilizer (kg ha^-1^).

$${\mathrm{AE}}_{N}\left(\mathrm{agronomic}\;\mathrm{efficiency}\;\mathrm{of}\;\mathrm{applied}\;N\right)\;\left({\mathrm{kg}\;\mathrm{kg}}^{‑1}\right)=\left(Y_{N}{‑Y}_{0}\right){/F}_{N}.$$

$${\mathrm{PFP}}_{N}\left(\mathrm{partial}\;\mathrm{factor}\;\mathrm{productivity}\;\mathrm{of}\;\mathrm{applied}\;N\right)\;\left({\mathrm{kg}\;\mathrm{kg}}^{‑1}\right){=Y}_{N}{/F}_{N}.$$

$${\mathrm{PE}}_{N}\left(\mathrm{physiological}\;\mathrm{efficiency}\;\mathrm{of}\;\mathrm{applied}\;N\right)\;\left({\mathrm{kg}\;\mathrm{kg}}^{‑1}\right)=\left(Y_{N}{‑Y}_{0}\right)/\left(U_{N}{‑U}_{0}\right).$$

#### 2.2.3 CO_2_, CH_4_ and N_2_O fluxes

**Fig 1:**
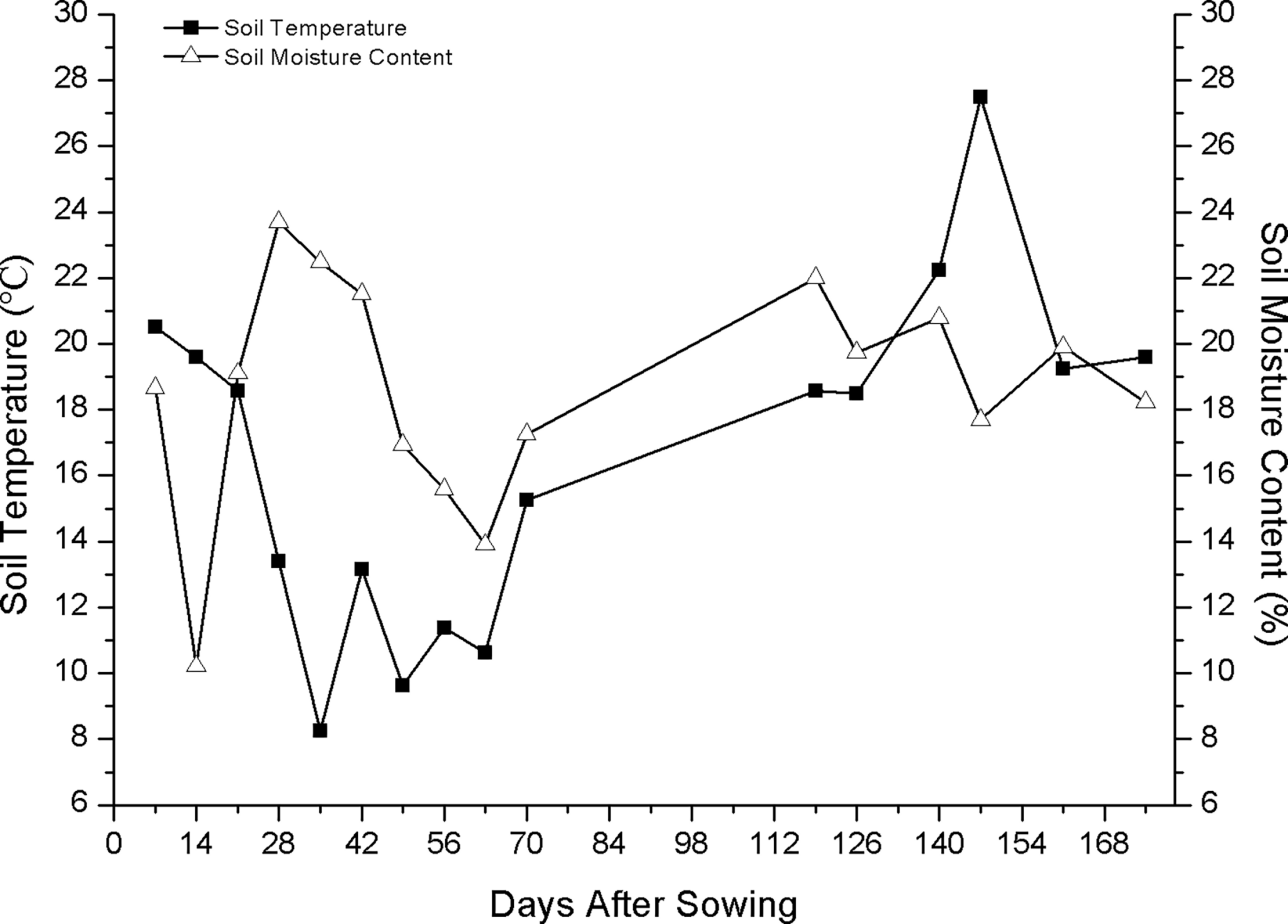
Soil temperature and soil moisture content during the 2014/2015 growing season.

After sowing, the dark static chamber/GC method was used to detect the CO_2_, CH_4_ and N_2_O fluxes between 9:00 am and 11:00 am every 7 days from November 9 to May 30 during the 2014–2015 season. At the same time, the soil temperature and soil moisture content were also measured (Fig 1 and S1 Table). The chamber covered a field area of 0.25 m^2^ and was placed on a fixed PVC frame located on each plot. The chamber was wrapped with a layer of sponge and aluminum foil to minimize the air temperature changes inside the chamber during the sampling period. The chamber was 0.5 or 1.1 m high and was adapted based on crop growth and plant height. Each sampling was subdivided five times in 10-min intervals. A fan was used to mix the gases in the chamber, which were then drawn off using a 20-ml gas-sampling syringe. The concentrations of CO_2_, CH_4_, and N_2_O were simultaneously detected using a gas chromatograph (Agilent 7890A, Shanghai, China) in the laboratory.

The increase in the GHG concentration in the static chamber was calculated by linear regression. Fluxes were calculated based on the following formula [35].

$$F=\frac{dc}{dt}\times \frac{mPV}{ART}=H\times \frac{dc}{dt}\times \frac{mP}{RT}.$$

Here, dc/dt is acquired from the linear regression equation. The value m is the molecular weight of trace gas, P indicates the atmospheric pressure (P = 1.013×10^5^ Pa), R is the gas constant (R = 8.314 J/mol/K), and T is the air temperature in the chamber. V, H, and A are the volume, height, and area of the static chamber, respectively.

Sample sets were rejected unless linear regression yielded an r^2^ value greater than 0.90. The seasonal CH_4_, N_2_O and CO_2_ emissions were sequentially linearly determined based on the emissions between every two adjacent intervals in the measurements. The air temperature inside the chamber was monitored during gas collection, and it was calibrated for the flux calculation.

The emission factor (EF-N_2_O) refers to the percentage of N that is released in the form of N_2_O to the applied N nutrients.

$${\mathrm{EF}‑N}_{2}O\;\left(\%\right)=\left(E_{N}{‑E}_{0}\right)/\mathrm{NAR}\times 100\%.$$

where E_N_ and E_0_ are the cumulative NO_2_-N emissions (kg N ha^-1^) from the fertilized and unfertilized plots, respectively, and NAR represents the N application rate (kg N ha^-1^)

#### 2.2.4. GWP and GHGI values

The global warming potential (GWP) of a greenhouse gas depends on its life time. Considering a time horizon of 100 years, the N_2_O and CH_4_ warming potentials are estimated to be 298 and 25 times higher than the CO_2_ warming potential, respectively [36]. The net global warming potential (net GWP) excluded CO_2_ [36].

$${\mathrm{GWP}=\mathrm{CO}}_{2}{+25\mathrm{CH}}_{4}{+298N}_{2}O.$$

$${\mathrm{Net}\;\mathrm{GWP}=25\mathrm{CH}}_{4}{+298N}_{2}O.$$

GHGI is related to grain yield, as described in Mosier et al. and Shang et al. [37, 38].

$$\mathrm{GHGI}=\mathrm{GWP}/\mathrm{grain}\;\mathrm{yield}\;\left({\mathrm{kg}\;\mathrm{CO}}_{2}{\mathrm{eq}\;\mathrm{kg}}^{‑1}\mathrm{grain}\;\mathrm{yield}\right).$$

### 2.3 Statistical analysis

Data were subjected to statistical analysis (ANOVA) using the IBM SPSS 21.0 statistical package (SPSS, 2012). Emissions of CO_2_, CH_4_, and N_2_O followed a logarithmic distribution, and log transformations of these emissions were used for statistical analysis. Significant differences among means were determined by Duncan’s multiple range tests at P ≤ 0.05. Pearson’s bivariate correlation analysis was used to evaluate the relationships between GHG emission and both yield and nitrogen utilization parameters.

## 3. Results

### 3.1 Grain yield and protein content

As seen from Table 2, in two crop years, the grain yields increased significantly due to the application of more nitrogen fertilizer; however, yields reached a plateau at 229.5 kg N ha^-1^, after which the wheat yield was hardly affected by the NAR. Compared to the HN plot, the grain yield in the MN plot was almost the same (2014/2015 growing season) or even higher (2013/2014 growing season); however, the NAR could be efficiently reduced by 15%. In contrast, the wheat yields in the two growing seasons significantly decreased by 13.5% and 13.0%, respectively, when the NARs were reduced by 30% in the LN treatment, which negatively affected wheat production. Similarly, yield responses were almost the same between the MN and HN plots, and both were significantly higher than that of the LN plot. The responses of grains per spike to the NARs were positive; however, there were no significant differences in the number of effective spikes among the different nitrogen application treatments. The improvements in grain yield were mainly due to the interaction of grains per spike and thousand-grain weight. The protein content increased as more N was applied in the 2014/2015 growing season, but the protein content was even higher in the MN plot than in the HN plot during the 2013/2014 growing season.

**Table 2:** Effects of different nitrogen applications on grain yields of winter wheat.

Year	Nitrogen rate (kg ha^-1^)	Spikes number per hectare (×10^4^ ha^-1^)	Grains per spike	1000 grains weight (g)	Grain yield (kg ha^-1^)	Protein content (%)	Yield response (kg ha^-1^)
2013–2014	0	316.7±16.9b	35.7±5.2c	38.9±0.3a	4256.6±136.7c	10.0±0.72c	-
	189	458.1±30.7a	38.8±7.1b	38.8±0.2a	6746.8±154.5b	13.6±0.1b	2490.2±154.5b
	229.5	485.2±12.5a	41.6±6.3ab	39.0±0.6a	7797.6±130.3a	14.7±0.10a	3541.0±130.3a
	270	477.8±11.1a	42.4±4.8a	38.8±0.6a	7715.9±305.1a	13.7±0.05b	3459.3±305.1a
2014–2015	0	302.7±11.2b	32.2±7.2c	43.5±0.5a	3860.0±351.6c	9.7±0.07d	-
	189	438.7±19.3a	38.7±9.2b	38.7±0.6d	6403.3±66.6b	13.5±0.35c	2543.3±66.6b
	229.5	449.7±17.9a	40.8±9.1ab	41.9±0.5b	7330.0±110.0a	14.4±0.28b	3470.0±110.0a
	270	456.3±15.6a	43.3±9.4a	40.0±0.9c	7360.0±151.0a	15.3±0.00a	3500.0±151.0a

Data are means ±standard deviation (SD) of six independent measurements, and different letters within a column indicate statistical significance at the *p* = 0.05 level using Duncan’s multiple range tests.

### 3.2 Nitrogen utilization parameters

As shown in Table 3, in the two crop years, the AE_N_ significantly increased by 20.3% and 16.2%, respectively, in the MN plot compared to the HN plot. Additionally, the AE_N_ of the MN plot was higher than that in the LN plot in both years. The PFP_N_ decreased significantly due to the increasing NAR, which reflected the law of diminishing returns. Thus, the 15% reduction in the NAR was an effective measure that improved the NUE without reducing the grain yield.

**Table 3:** Effects of different nitrogen application rates on nitrogen utilization parameters.

Year	Nitrogen rates (kg ha^-1^)	AE_N_ (kg kg^-1^)	PFP_N_ (kg kg^-1^)	AR_N_ (%)	PE_N_ (kg kg^-1^)
2013–2014	189	13.2±0.82b	35.7±3.30a	46.7±0.57b	28.2±1.75a
	229.5	15.4±0.57a	34.0±3.06b	58.4±0.91a	26.4±0.97a
	270	12.8±1.13b	28.6±1.13c	59.4±0.69a	21.6±1.90b
2014–2015	189	13.5±0.35b	33.9±0.35a	43.5±0.44c	30.9±0.78a
	229.5	15.1±0.48a	31.9±0.48b	54.6±2.04b	27.7±0.91b
	270	13.0±0.56b	27.8±0.56c	58.2±0.47a	23.1±0.87c

AE_N_: agronomic efficiency of applied N fertilizer; AR_N_: apparent recovery efficiency of applied N fertilizer; PFP_N_: partial factor productivity of N fertilizer application; and PE_N_: physiological efficiency of applied N. Data are means ±standard deviation (SD) of four independent measurements, and different letters within a column indicate statistical significance at the *p* = 0.05 level using Duncan’s multiple range tests.

AE_N_ can be further decomposed into the AR_N_ and PE_N_ of applied N. The AR_N_ improved as the NAR increased, while the PE_N_ was negatively affected by the increase in the NAR. In the 2013/2014 growing season, the AR_N_ in the MN plot only had a slight reduction of 1.7%, which was not significant relative to the HN plot; however, the PE_N_ in the MN plot was significantly higher than that in the HN plot.

### 3.3 Greenhouse gas (GHG) emissions, net GWP, and GHGI

#### 3.3.1 GHG emissions

As seen in Table 4, the CO_2_ released from soil and plants was the largest source of greenhouse gas emission in all treatments. It was observed that the cumulative CO_2_ emissions significantly increased with increases in the NAR during the 2014/2015 wheat growing season. There was no obvious relationship between the CH_4_ emissions and NAR, and the lowest cumulative emissions were measured in the MN plot. The cumulative N_2_O emissions gradually increased with increases in the NAR, and the values varied from 0.621 to 1.32 kg N ha^-1^, which were equivalent to 0.41%-0.48% of the N fertilizer that was applied. Relative to the HN plot, the seasonal N_2_O emissions significantly decreased by 19.1% in the MN plot. The MN practices emitted 16.6% more N_2_O because they received additional N through the higher application relative to the LN treatment; however, this difference was not significant. The emission factor (EF-N_2_O) relative to the applied N was measured to range from 0.144 to 0.258% in all nitrogen treatments. Compared to the HN plot, the EF-N_2_O decreased by 39.9% and 24.8% in the LN and MN plots, respectively.

**Table 4:** Total emission of greenhouse gas during whole growth period of winter wheat.

Nitrogen rate (kg ha^-1^)	CO_2_ (kg ha^-1^)	CH_4_ (kg ha^-1^)	N_2_O (g ha^-1^)	EF-N_2_O (%)	Net GWP (kg ha^-1^)	GHGI
0	10841.0±265.7d	5.84±0.11b	621.0±18.0c	-	331.0±2.6c	2.89±0.07a
189	16262.3±489.9c	4.80±0.23b	914.1±80.4b	0.155±0.021c	392.3±18.3b	2.60±0.07d
229.5	18067.0±230.7b	2.75±0.09c	1065.8±97.9b	0.193±0.021b	386.3±26.8b	2.52±0.04de
270	19896.1±503.4a	9.57±0.05a	1317.5±32.0a	0.258±0.006a	631.8±8.4a	2.79±0.07ab

EF-N_2_O: the emission factor refers to the percentage of N that is released in the form of N_2_O to the applied N nutrients. Net GWP: net global warming potential. GHGI: greenhouse gas intensity. Data are means ±standard deviation (SD) of six independent measurements, and different letters within a column indicate statistical significance at the *p* = 0.05 level using Duncan’s multiple range tests.

#### 3.3. 2 Net GWP and GHGI

**Fig 2:**
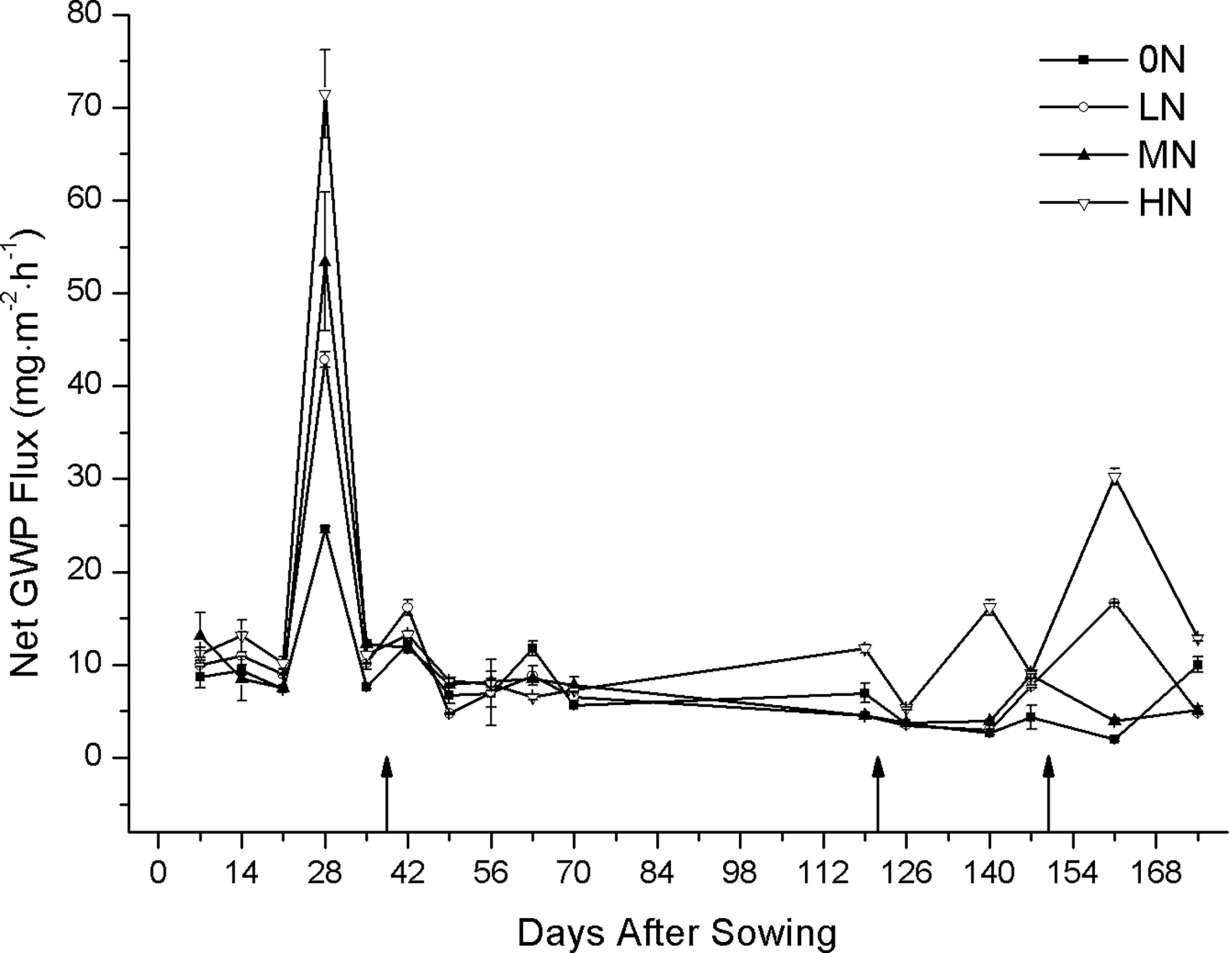
Seasonal net GWP flux during 2014/2015 growing season. 0N: 0 kg N ha^-1^; LN: 189 kg N ha^-1^; MN: 229.5 kg N ha^-1^; HN: 270 kg N ha^-1^. Standard deviation (SD) is denoted by error bars. Arrows in the figure indicate the top-dressing time.

The seasonal net GWP flux during 2014/2015 growing season was presented in Fig 2 and S2 Table. Higher net GWP fluxes occurred in the early of growing season, and the highest peak of net GWP fluxes were recorded at the 28 days after sowing. Peak net GWP flux increased with NAR, with rates ranging from 24.56 to 71.47 mg N m^-2^ h^-1^. The net GWP flux under all treatments were concentrated on the sowing-before wintering stage, accounting for 41.0–49.7% of total emissions from the whole wheat growth period. There were several small emission peaks in the next days. After stem elongation, the period of rapid wheat growth, fast uptake and utilization of soil N occurred, resulting in the slight peak of net GWP flux in the week after top-dressing under all N application conditions. At later stages, large amounts of soil N were absorbed and utilized for wheat growth along with rising temperature, leading to minor changes in net GWP under all N conditions.

Although CH_4_ emissions were not obviously affected by N fertilization, N_2_O emissions significantly increased as N increased. Accordingly, the net GWP significantly increased with the increase in the NAR. Relative to the net GWP (631.8 kg CO_2_ eq ha^−1^ yr^−1^) from the HN plot, the net GWP was reduced by 61.1% and 62.1%, respectively, in the LN and MN plots. Compared to LN plot, the net GWP value was even lower than that in the MN plot.

The lowest GHGI was observed in the MN plot, which was 12.8% lower than the value in the 0N plot and 9.7% lower than the value in the HN plot. Hence, the MN practices provided ecological solutions for wheat production and, therefore, deserve considerable attention.

### 3.4 Correlation analysis between GHG emission and nitrogen utilization parameters

The results of correlation analysis between the nitrogen utilization parameters and the GHG emission index are shown in Table 5 and S3 Table. The N_2_O emission was negatively correlated with PFP_N_ (r = - 0.999*, p < 0.05) and PE_N_ (r = - 0.999*, p < 0.05), indicating that a reduction in N_2_O emissions improved the PFP_N_ and PE_N_ to some extent. Similarly, both CO_2_ and CH_4_ emissions were negatively correlated with the PFP_N_ and PE_N_, and the trends were similar to those of the N_2_O emission. All these findings resulted in a negative correlation between the net GWP and the PFP_N_ (r = - 0.940, p > 0.05) and PE_N_ (r = - 0.904, p > 0.05). Furthermore, a negative correlation was also observed between the GHGI and the AE_N_ (r = - 0.865, p > 0.05), PFP_N_ (r = - 0.814, p > 0.05) and PE_N_ (r = - 0.756, p > 0.05).

**Table 5:** Correlation analysis between GHG emissions and nitrogen utilization parameters.

	CO_2_	CH_4_	N_2_O	EF-N_2_O	Net GWP	GHGI
AE_N_	-0.232	-0.868	-0.364	-0.371	-0.700	-0.865
PFP_N_	-0.982	-0.811	-.999*	-0.999*	-0.940	-0.814
AR_N_	0.958	0.447	0.910	0.906	0.674	0.451
PE_N_	-0.995	-0.753	-0.999*	-0.999*	-0.904	-0.756

* indicate significance of *r* values at *p* = 0.05 by Pearson’s bivariate correlation analysis, n = 3.

## Discussion

### 4.1 Comparison of grain yield, AE_N_ and N_2_O emission in wheat grown under 229.5 kg N ha^-1^ and 270 kg N ha^-1^

N application cannot promise a substantial increase in crop productivity due to the principle of diminishing returns [39]. Our grain yield under the application of 229.5 kg N ha^-1^ was as high as that under 270 kg ha^-1^ N level, which was accordant with previous studies [40, 41]. N application amount to winter wheat in Wuxi County has now been reduced by 20–45 kg N ha^-1^ or 10–20% without concomitant yield decreases, from formerly around 230 kg N ha^-1^ [42, 43]. Additionally, synthesized from an economic and ecological point of view, 150–225 kg N ha^-1^ is recommended in southern China [44]. Furthermore, significant increase (20.3%) of AE_N_ was reported under 229.5 kg N ha^-1^ condition relative to 270 kg N ha^-1^ in our study, which were concordant with previous results that AE_N_ gradually increased with N reducing [45, 46]. Thus, a quantum leap in the AE_N_ is possible by simply reducing the N rate to 229.5 kg ha^−1^ in winter wheat; this reduction is primarily possible because the high N inputs of 270 kg N ha^−1^ in this region are excessive, and not all nitrogen is absorbed. The cumulative N_2_O emission gradually increased with N increasing in this paper, which was consistent with previous studies [47, 48]. Relative to 270 kg N ha^−1^, the seasonal N_2_O emissions was significantly decreased by 19.1% under 229.5 kg N ha^-1^ condition. Previously, a 10–30% reduction in N fertilizer would decrease N_2_O emissions by 11–22% in wheat [49], which were comparable with our findings. Meanwhile, N fertilizer reduction can lead to GHG emission reductions according to Kahrl's estimation [50]. Controlling the overall N rates to meet the needs of crop growth can help minimize N losses through N_2_O emissions. Hence, it is suggested that N_2_O emissions from wheat production can be reduced under the condition of 229.5 kg N ha^-1^ without yield reduction while AE_N_ improved significantly. These findings demonstrated that the N loss could be greatly reduced due to the increased crop uptake.

### 4.2 Comparison of N_2_O emission, grain yield and AE_N_ in wheat grown under 229.5 kg N ha^-1^ and 189 kg N ha^-1^

N_2_O is produced naturally in the soil through nitrification and denitrification and depends on soil mineral N contents [51]. The input of N fertilizers into agricultural systems is considered to be the dominant source of N_2_O emissions from agricultural soils [52, 53]. Since there exits significant correlations between N_2_O emissions and the amount of N applied [47, 48], N_2_O emissions gradually increased as more N input. However, in this study, no significant difference was observed on N_2_O emissions between 229.5 kg N ha^-1^ and 189 kg N ha^-1^. Hence, the practices of 229.5 kg N ha^-1^ provided ecological solutions for wheat production, therefore it deserved considerable attention. Grain yield was negatively affected with significant decrease under 189 kg N ha^-1^ condition compared to 229.5 kg N ha^-1^, which was consistent with previous studies [40, 41]. Based on the results of other studies, the AE_N_ declines when rates exceed 150 kg N ha^-1^ [54]. However, the highest values were achieved when 229.5 kg N ha^-1^ was applied. Although our highest AE_N_ (15.4 kg kg^-1^) was greater than the mean AE_N_ for winter wheat in China (9.4 kg kg^-1^), as reported by Chuan et al. [32], it was still lower than the world average AE_N_ for cereal crop production, i.e., 18 kg kg^-1^, as calculated by Ladha et al. [55]. It indicated that there still exists great potential for grain yield under the condition of 229.5 kg N ha^-1^. Generally, significant improvement in wheat production and AE_N_ were achieved with fewer potential threats to environment and ecology under 229.5 kg N ha^-1^ condition.

### 4.3 Comparison of AR_N_ and PE_N_ in wheat grown under three nitrogen Levels

Reported by previous studies, AR_N_ and PE_N_ were negatively affected as increasing N input [11, 12], which reflected the principle of diminishing returns. It is well known that the PE_N_ is very important to the AE_N_ because improvements in the PE_N_ directly result in greater plant biomass or grain yields. Normally, the stimulation effects of N fertilizer on PE_N_ dramatically reduced due to excessive nitrogen fertilizer. An interesting finding showed that under 229.5 kg N ha^-1^ condition, the AR_N_ was as high as it under 270 kg N ha^-1^, but the PE_N_ was significantly higher, indicating that the N absorbed by the plant was utilized more efficiently under 229.5 kg N ha^-1^ condition. Though a slight reduction in PE_N_ was found under 229.5 kg N ha^-1^ condition relative to 189 kg N ha^-1^ condition, the AR_N_ was significantly increased, which explained that more N could be absorbed from soil under 229.5 kg N ha^-1^. All these findings illustrated that wheat plant could uptake and utilize more efficiently under 229.5 kg N ha^-1^, which was the reason why the highest AE_N_ was achieved under 229.5 kg N ha^-1^.

## Conclusions

Grain yields increased significantly due to higher nitrogen fertilizer input, but reach a plateau at 229.5 kg N ha^-1^, in which wheat yield is hardly affected by higher nitrogen input. Meanwhile, under 229.5 kg N ha^-1^ condition, the highest AE_N_ value were achieved, and N_2_O emission was as low as that of 189 kg ha^-1^ N. These findings demonstrated the practice of 229.5 kg N ha^-1^ could be identified as a fertilizer threshold which was conductive to enhancing the sustainability of crop production in our research region. Whether it is also beneficial for other region or other cultivar is still remains unexplored.

## Supporting information

S1 Table Soil temperature and soil moisture content during the 2014/2015 growing season. (XLSX)

S2 Table Seasonal net GWP flux during 2014/2015 growing season. (XLSX)

S3 Table Effects of different nitrogen applications on grain yields, nitrogen utilization parameters and GHG emissions of winter wheat. (XLSX)
